# μετὰ τὰ ϕυσικά: Vision Far Beyond Physics †

**DOI:** 10.3390/vision9020025

**Published:** 2025-03-26

**Authors:** Liliana Albertazzi

**Affiliations:** Retired from Trento University. Current address: Via Ca’ La Selva, 4, 47855 Gemmano (RN), Italy; lili.albertazzi@gmail.com

**Keywords:** appearances, De Chirico, experimental phenomenology, imagination, metaphysical painting, past experience, pictorial art, visual thinking, virtual reality

## Abstract

Vision Science is an area of study that focuses on specific aspects of visual perception and is conducted mainly in the restricted and controlled context of laboratories. In so doing, the methodological procedures adopted necessarily reduce the variables of natural perception. For the time being, it is extremely difficult to perform psychophysical, neurophysiological, and phenomenological experiments in open scenery, even if that is our natural visual experience. This study discusses four points whose status in Vision Science is still controversial. Namely, the copresence of distinct visual phenomena of primary and secondary processes in natural vision; the role of visual imagination in seeing; the factors ruling the perception of global ambiguity and enigmatic and emotional atmosphere in the visual experience of a scene; and if the phenomena of subjective vision are considered, what kind of new laboratories are available for studying visual perception in open scenery. In the framework of experimental phenomenology and the use of pictorial art as a complement and test for perceptual phenomena, a case study from painting showing the copresence of perceptual and mental visual processes is also discussed and analyzed. This has involved measuring color and light in specific zones of the painting chosen for analysis, relative to visual templates, using Natural Color System notation cards.

## 1. Introduction: The What, the How, and the Why in Vision

From its inception, Vision Science has built its credentials as an exact science on the development and testing of theories and research hypotheses, experimental methodologies, model building, objectivity of results in third person accounts validated by statistical analyses, correlational or causal explanations, and the idealization of a universal perceiver [1,2]. The general aim of Vision Science has always been to answer questions such as what do we really see, how do we see, and why things appear to us as they do [3]. However, in the past century the answers given to these questions have been notably different and sometimes in total opposition to each other. One example of this is the incompatibility in recent decades between the ecological [4] and the inferential approach to vision based on unconscious inferences [5,6], even if the variety of proposals is much wider due to the increasing significance of computational models [7]. What has almost never been questioned by these different approaches is the role of physics [8] as the foundation of the experimental psychological research on which classic psychophysics and neuroscience have based their scientific justifications. When this assumption has had to account for eccentricities not foreseen by the reference structure, as in the case of so-called perceptual illusions, a wide variety of phenomena deviating from the laws of optics and more generally in contradiction to the distal stimuli, have usually been classified as perceptual errors. There has been no universal explanation of these phenomena either [9,10,11,12,13], even if references have often been made to top-down factors such as interpretations, probabilistic assumptions, past experience, and so on. More in general, physics has remained the root explanation for the occurrence of the facts of vision, while the meaning of visual phenomena has been ascribed to the intervention of higher-level cognitive functions. Given the premises, this is an understandable compromise, considering that in physics, there is no meaning.

Breaking away from this general framework, and questioning the accredited premises of vision research, Gestalt psychology [3,14,15,16] and, later, experimental phenomenology (see [7,17]) have marked a change in direction, founded on an alternative theory of Aristotelian origins, descriptive and demonstrative methodologies, and experimental methods based on the objectification of first person experiences. In so doing, the phenomenological approach to vision gives a very different response to the main questions mentioned above, by making appearances the object of the science of vision (the what) [14,18], and developing the principles that allow and explain their emergence (the why) in awareness [19,20]. With regard to the way objects of vision appear (the how) [18,19,21], which is one of its main achievements, the phenomenological approach has built an entire archive of appearances on both a descriptive and an experimental basis. Thanks to this radical change, experimental phenomenology has gone beyond physics, revising much of the scientific methodologies and common concepts in vision science. Think, for example, of the concept of presentation (i.e., *Vorstellung*) [22] (pp. 3–5) (see also [23]) which has replaced the generally unquestioned concept of the visual representation of physical stimuli. A presentation in awareness (“presence”) consists of phenomenal appearances and their variations and inner relationships, given in subjective anisotropic space and a subjective time duration, which are dimensions of awareness structurally beyond the field of physics. This concept of Aristotelian origin (Φαντασία, phantasia) [24] (p. III.3) refers to an inner process of “what makes visible or appear”, whose outcomes are appearances (*phantasmata*) devoid of physical matter. One may say that phantasia generates appearances in awareness from within: in this sense, phantasia is neither reproductive nor a function of high-level cognition. This process is also at a crossroads between, on one hand, the sensory perception triggered by physical stimuli, and, on the other, the high-level mental field (διάνοια) of conceptualizations (*noemata*). It is worth noting that the concept of phantasia includes any kind of appearances in awareness, i.e., in perception, dreams, fancy, hallucinations, and illusions (similarly, see [18,22,25,26]). Vice versa, the concept does not include any kind of thinking, such as reasoning, inferences, true or false judgments, or beliefs that require other psychic functions (νοῦς). Therefore, the layered richness of the Aristotelian concept does not wholly coincide with the wider modern meaning of imagination, and can be defined as “primary imagination” (in this tradition and with regard to vividness as an intensive aspect of the conscious experience of the perceptual presentations, see [27]). More specifically, the original concept does not coincide with the following points:

1. Imagery as a literary device;
2. Imagery as an internal representation used in formation processing, where depictive representations rely on topographic organized regions of the brain [28,29];
3. Mental operations on images as pictures of physical objects [30];
4. Recreations of an experience that resemble what is actually perceived, like a depictive representation [28,31];
5. Images that rely on depictive representations interpreted by other mental processes or directly conceived as propositional representations in computer models of imagery;
6. Imagination as a top-down constraint, a sort of cognitive penetration of perception [32,33].

Notwithstanding their differences, the above-mentioned contemporary approaches to the so-called imagery debate share a common ground—the concept of mental representation of physical objects—and refer to either lower (the underlying brain functions) or higher mental levels of object representation. The Aristotelian concept of phantasia, on the other hand, concerns the qualities of the subjective experience itself, as the images are what presently appear to awareness (appearances). This approach became a cornerstone of phenomenology and Gestalt psychology. What the Aristotelian concept may share with modern approaches, despite its many differences, is the role of imagination in visual perception [28].

### Primary and Secondary Processes and the Role of Past Experience

To ensure the objectivity and meaning of perceptual phenomena in awareness, experimental phenomenology has carried out extremely accurate investigations of the characteristics of appearances (the way), their classification [15] (p. 14ff), and their empirical and experimental demonstration. In this field, Kanizsa achieved a well-known result, using an Aristotelian concept, that heuristically demonstrates the difference between primary and secondary perceptive processes. The first are characterized by the real “presence” of the phenomena given in awareness, i.e., the phenomena caused by perceptual integration and not reflective thinking (the most striking example being amodal completion [21,34]). The second are mental processes characterized by the visual data being enriched by inferential and/or abstraction operations, hypotheses, memories, logical principles, rational categorizations and probabilistic calculations [35] (pp. 26–30). To perform the latter, incidentally, does not require the presence of a visual perception (see also [36]). Kanizsa’s empirical demonstrations showed that the two processes—although co-present and practically inseparable in seeing—are different, follow different rules, and need different explanations. But Kanizsa was primarily interested in the autochthonous factors of perceptual organization (the grammar of seeing), especially in object formation, depth, order, and motion [21]. He was not interested in factors potentially affected by past experience, affective valence and meaning [37]; the (micro)genesis of appearances [38,39] (see also [40]); and least of all, the general simultaneous presence of the two processes in the visual experience of a natural scene which implies a degree of indeterminacy (in “the world”, as Kanizsa provocatively expressed it). He focused on the perceptual organization of specific visual phenomena, such as phenomenal margins in the absence of stimulation discontinuities, amodal completion, phenomenal transparency, intentional movements, and so on. This left several questions in the background, like the inner dynamic relationships between primary and secondary processes; for example, the presence of forms of visual categorization (the perception of generic qualities or “types”, such as circles or square, see [41]) to which high order processes apply, and whether the perceptual laws of organization are also active in the secondary process [35] (Ch. 2) (see also [42]). With regard to the possible explanation of the relationship between primary and secondary processes, Kanizsa noticed the plurality and disparities of theories and hypotheses, concluding that it continued to be an unresolvable and somehow mysterious problem [35] (Ch. 3). Briefly, and still to be verified, there is the question of whether there is a leap or continuity between the primary and secondary processes; how and on what grounds a perceptual presentation is connected with a mental representation; and the role of imagination in any kind of productive thinking [43]. As Wertheimer observes, to classify thinking as a separate chapter of psychology, detached from human attitudes and emotions is an artificial and narrow view [43].

The sharp distinction between primary and secondary processes led to a disagreement between Kanizsa and Musatti, who maintained that it was impossible to experimentally demonstrate a clear distinction between the natural factors of perceptual organization and empirical assimilative factors (the so-called impossible experiment) [44]. Musatti [45,46], in fact, identified the role of past experience in terms of assimilative and field factors that modify, transform and enrich the perceived phenomenon. In both cases, the role of past experience in primary processes was shown to be due to empirical field factors and not merely individual memories or inferences. This surely weakens the importance given to the concept by probabilistic, inferential, and cognitive approaches to vision (regarding the priority of the laws of organization on past experience see also [18] (Ch. 1, 11)).

Research in the Gestalt tradition is hardly comparable or transposable to a different approach, like the Bayesian tradition, which is valuable when applied to cognitive/secondary processes, especially when a judgment is required, but not an adequate tool for the analysis and explanation of primary processes and subjective perception (see [47,48,49]). More specifically, models of inference consider the main task of perception to be the recovery of a perceptual “representation” and the processing of information already manifest at the physical level, which is subsequently and unidirectionally uncovered and elaborated at the sensory and neuronal level. Experimental phenomenological and inferentialist approaches have a different significance and applicability in Vision Science and differ greatly regarding the concept of past experience. In experimental phenomenology, perception is not considered to be the product of an unconscious inference process, a calculation, or the result of psychophysical or neuroscientific knowledge; it is a direct acquisition of the qualitative information provided in awareness. It is worth noting, then, that when discussing the influence of the past on perception, Köhler also distinguished between an imaginative dimension similar to memory, but not in the common sense of the term [50] (Ch. 7).

An interesting insight on the thorny issue of past experience can be found in Karl Duncker’s studies of induced movement [51], which are based on pictures of visual objects previously experienced as moving or static. Duncker demonstrates a tendency towards unity, centralization of the system of reference, and maintaining reference relationships. The role played by experience in perception is identified in the influence the situational configuration of an object/event has on actual perception and determining the degree to which a visual object acquires new properties (and specifically, tertiary qualities). For example, the mere image of a lighthouse, which is known to be relevant to a certain marine setting and related to the movement of ships, tends to reproduce this setting and influence other perceived objects.

It is worth noting that today, the way phenomena (from both primary and secondary processes) behave out of the restricted and controlled situation of the lab remains largely untested. In fact, it is quite difficult to perform psychophysical or neurophysiological experiments in open scenery, which nevertheless is the natural experience for the subject. Furthermore, like the other sciences of perception, experimental phenomenology also performs its experiments in controlled protocols and conditions, which necessarily reduce the variables of natural perception. For the time being, then, we have only laboratory research. What one can say is that in our daily experience, secondary processes, although distinct, are rarely absent. This is particularly evident in motor and intentional tasks like visiting a museum, a place of worship or historical ruins or simply going shopping or mushroom picking.

## 2. My Contribution

In this paper, I will address several aspects of vision whose status is either still controversial, like the nature and role of past experience in perceiving, or rarely addressed, like the role of imagination and the factors ruling the perception of global ambiguity and enigmatic and emotional atmosphere in the visual experience of a scene.

A good example of the complexity of this issue and its visual description is offered by pictorial art, a field from which I will also discuss a case study: De Chirico’s metaphysical painting. This choice has been made for the following reasons. The closeness of phenomenology to the esthetic dimension has been noted since the dawn of phenomenological experimental research. The Wertheimer laws of organization are very similar to the basic principles of design [52]; Metzger highlighted the close relationship of art and perception on several occasions [18]; Kanizsa considered pictorial art to be a test bench for perceptual phenomena and vice versa [35] (p. 83), and his profound belief was that pictorial art and experimental phenomenology complement each other in terms of mutual integration. Moreover, he was himself the author of a series of morpho-biological drawings (https://www.gaetanokanizsa.it/home-en/, accessed on 12 March 2025). As for Massironi, both his experimental and artistic work are detailed demonstrations of the laws of organization in vision (https://www.artnet.com/artists/manfredo-massironi/, accessed on 12 March 2025). On the other hand, the experience of apprentices in art academies, like the Bauhaus, also turned out to be more a question of learning to see than of learning a mere technique (cit. [53]). The growth in contemporary literature on art and visual perception [54], the establishment of new Journals (*Art & Perception*, Brill) and Centers (like CSPE in Glasgow: https://www.gla.ac.uk/research/az/cspe/about/, accessed on 12 March 2025, and the newly established CArPe in Padua: http://www.dpg.unipd.it/en/carpe, accessed on 12 March 2025), and the many conferences on the topic are a sign of the lively research being conducted in this field. In fact, the visual analyses performed by artists are important explorations of the phenomenology of vision and real sources for visual theory, as they unveil the structure of the subjective visual space elements and demonstrate the conditions that make appearances visible. Last but not least, phenomenological experimental research, which remains close to nature [18] (p. 181ff) and [18] (Ch. 12) as we subjectively experience it, has a potential which no other scientific approach to vision seems to share. It can therefore be used as a tool by both designers and researchers working in the fields of computer graphics, Augmented Reality, and Virtual Reality (see Section 10).

This contribution considers the astonishing capacity of painters to visualize dimensions of the subjective perceptual experience that still need a proper classification, explanation and experimental verification [55,56]. Of all the modern painters, De Chirico is of particular interest to Vision Science, because his aristocratic, refined, and allusive painting visualizes in plastic forms the grounding role of imagination, and the presence of affective valence and meaning in the visual perception of environmental wholes. In so doing, his painting crosses and shapes several levels of psychic processes and reveals the pervasive role of imagination in visual perception.

The paper therefore progresses through the following sections:

3. Art and visual perception;
4. The case study: Metaphysical art;
5. The what: Visual reality;
6. The how: Means to illusoriness;
7. Empirical analysis: *La matinée angoissante;*
8. Discussion: Relevant outcomes;
9. Conclusions: The role of imagination in a world of appearances;
10. Future directions: Laboratories for visual imagination.

For anyone who is interested, further information on De Chirico’s work is provided at the end of this paper (Appendix A: *De Chirico, an idiosyncratic avantgarde painter*). The paintings considered are mentioned with their original titles and a translation in square brackets. Most of De Chirico’s paintings, and especially those considered, are visible and available on the web. Special attention has been given to the *La matinée angoissante* [The anguished morning] (1912), due to the colorimetric analysis I conducted personally on the painting with the NCS Atlas at MART (Museum of Modern Art) (see Section 7).

## 3. Art and Visual Perception

The relationship between visual perception, scientific knowledge, and art spans the entire history of art [57], involving both the chemistry of materials for rendering the textures of the appearances [58], and the representation of visual space in different perspective modalities [18,41,55,59,60,61,62]. This topic has also raised the interest of original physicists such as Bohm [63] (Ch. 2).

It is known, for example, that in Western painting, the transition from tempera to oil shifted the lightness and transparence of appearances to a darker tint, and that achieving the brilliance and naturalness which characterizes, for example, the appearance of still life was due to the intense craftsmanship of artists like the Flemish School (see for example, Willem van Aelst’s paintings). As for the spatial placement and behavior of the pictorial forms on the canvas, the transition from a sort of two-dimensional grid organized in vertical and horizontal lines to an isometric perspective with the insertion of parallel lines that run obliquely through the spatial structure does succeed in promoting a perception of depth but also loses a clear center in favor of apparent movement along the horizontal plane (see below, Section 6).

A classic example of the time dimension intrinsic in spatial perception is given by traditional Japanese scroll painting, which conveys the perception of a silent narrative and allows the viewer to travel virtually through a landscape (for the representation of time in painting see [64]). This type of visual perception reveals many aspects that also characterize natural vision, such as the presence of simultaneous vistas in landscapes (see Kano Hideyori, *Shihoncho shokkanpūzu* [Maple viewers], 1565–1576)). The perception conveyed by the open scrolls is just one case in a series of much stronger perceptions of illusory movements, like the phi phenomenon [65], all of which have a subjective time/space structure of awareness [40].

The use of different and contradictory perspectives in the same painting also conveys an inevitable feeling of ambiguity and disorientation, as the violation of perspective rules allows multiple spaces and viewpoints to be depicted in the same scene. In this respect, painting offers a series of clues regarding the nature of perceptual concept formation (what Arnheim defined as “visual thinking” [41]; see also [66] (p. Ch. 2)). Visual thinking (a process) is the ability to grasp certain prominent characteristics of a perceived object (the rectangularity of a book or a building, the blue of the sky, the outline of a face), i.e., the salient structural configurations (“types”) that make up subjective perception. What the artist does is to create patterns and configurations that visualize the meaning of experience through perceptually organized forms [66]. Lipps [67] anticipated much of the subsequent experimental research on visual perception and empirical and experimental esthetics by showing that both anisotropy and certain characteristics of visual space, including the role of its “geometric” visual elements (points, lines, circles, squares), their configurations (lines in the angles, for example), and their visual distortions [68] are intrinsic. In short, the perception of pictorial works of art is a source of information for several aspects of vision (see, for example [68,69]).

A thorough analysis of the intrinsic relationship between pictorial art and visual perception, the behavior of shapes, and the role of visual elements placed, displaced, organized and molded by the invisible perceptual forces in this field, can be found in Arnheim’s work [66], which is a genuine treatise on the demonstrative methodology of experimental phenomenology in vision [54,70,71,72]. Over the centuries, there have been many variations and examples of the close relationship between art and perception, indicated in Arnheim’s work. By choosing non-figurative painting, for example, artists highlight the relevant dimensions of the primary process of vision and the elements of seeing [73]. These include the visual, compositional strength, the expressiveness of the line and its ways of appearing, as well as the perceptual object, hatching, and outline (above all [74]. Other examples of these percepts (see [71,75,76]) are as follows: primitive geometrical shapes, such as triangles, rectangles and monochrome squares that mold visual things (Malevič); three-dimensional, symbolic, and connotative properties of color (Marc); the expressiveness of shapes (Kirchner, Schiele); space–time deformations, perspective variations, and the dynamism and continuity of forms in space (Boccioni); the exaggerated use of polygons by Futurism (Picasso, Braque); and accentuation of the perception of reality as it is subjectively expressed by the Fauvists (Matisse).

In addition to clarifying that pictorial presentations *are* themselves a modality of perceptual awareness [59,73,77], the development of art movements also highlighted and conveyed the presence of meanings relating to visual perceiving, such as, the awareness of ambiguities, the perceived continuity in awareness’ phenomenal degrees, the often indecipherable presence of atmosphere and cultural influences. Examples of the latter include the conflicting traces of religion that remain in Kandinsky’s journey to abstractionism [78], the Hebrew roots and imaginative folk scenarios in Chagall’s work, the Byzantine light in Klimt’s use of gold, and the social and political issues in Futurism, not to mention the indissoluble entanglement of images and text in the three-dimensional appearance of Japanese scrolls. From this point of view, the avant-gardes and abstract art of the 20th century are an important chapter in the relationship between art and visual perception, because they clarified and shaped certain fundamental characteristics of the complexity of vision in primary and secondary processes.

What matters for our purposes is how research into painting aligns with and helps shed light on the nature and behavior of the different dimensions of appearances, classified by Metzger [15] as ranging from those having a more solid character or reality (to be “real”) to those perceived with a lesser degree of reality, like shadows [79], points of light [19,80], mirror images [81,82], pictorial images, dreams, and so on. What remains unfocused in Metzger’s classification is the perception of atmosphere, like the experience of foreboding and estrangement embedded in the global visual scene. In these cases, the role of imagination and the psychic processes present in the vision of a reality, which cannot be reduced to physics, surfaces more and more forcefully. From this point of view, painters can visualize imaginative and emotional dimensions embedded in complex subjective experiences, using pictorial devices like the ones employed in De Chirico’s painting (see below, Section 6). Boccioni, for example, used force lines with an accentuated expressive charge to enter a space–time context; Rothko’s abstract paintings, whether in single colors or with blurred edges (think of the fourteen paintings in varying hues of black at the non-denominational Chapel in Houston) convey an ineffable atmosphere of deep sadness; and through their varying appearances of light, Turrell’s Skyspaces and the Roden crater are examples of both perception and self-awareness in seeing [77] (pp. 292–294). As for the visual genesis of appearances in awareness, consider, for example, Pepperell’s paintings (https://robertpepperell.com, accessed on 12 March 2025).

## 4. The Case Study: Metaphysical Art

De Chirico’s “Metaphysical art” (1908–1910), which he developed over many years, was triggered by an unexpected, surprising, and unsettling visual experience of the Florentine square of Santa Croce during the day and under a dense, low sky [83] (p. 377). He was shocked by what he defined as the plastic appearance of the scenery, its solidity and apparent changelessness, in a psychic state of suspension of time [83] (p. 230). As he later wrote, he had the strange impression of looking at things for the first time and the picture’s composition was formed in his mind’s eye immediately. This enigmatic experience triggered his metaphysical painting. In this perception, there were no subjects, he commented, because the sudden holistic visual appearance of the place was totally ante-predicative. In other words, the perceiver was an integral part and a functional component of the percept. This is by no means a surprise, as the state of emotional upheaval described by Stendhal after a visit to the Church of Santa Croce, which led to this syndrome being named after him, is a similarly extreme manifestation of the emotional impact that the visual experience of a location with a particularly rich cultural content can provoke. Just think of the stunning sense of amazement you feel when you suddenly find yourself in front of the baptistery in Florence. An effect that the distance of the oblique vanishing points from the sides may well contribute to (see [61] (p. 1268)).

Travelers often experience something similar when visiting cities like Florence and Turin, the cities of De Chirico’s paintings, as well as Ferrara (“the city of wonders”). On the other hand, what De Chirico describes is only the awareness of an “immediate datum”, even if that may appear “unusual, unexpected, illogical or meaningless, and even contradict indisputable axioms or familiar habits of thought” [15] (p. 12). As to the choice of the term metaphysical, as he often repeated, “I don’t see anything dark in the word “metaphysics”: it is the same tranquility and senseless beauty of matter that appears to me as “metaphysical” and those objects that appear even more metaphysical to me, due to their clarity of color and accuracy of measurements, are the antipodes of any confusion and any nebulosity” (something he accused Impressionist painting of) [83] (p. 172). De Chirico points to the primary foundations of a great metaphysical esthetic “in the construction of cities, in the architectural form of houses, squares, gardens, ports, railway stations” [83] (pp. 180–181). According to him, the “inside of a arch, the corner of a street or even the interior of a room, or the surface of a table, between the sides of a box” are embedded in a metaphysical alphabet (the “types”) and have an emotional aspect [83] (p. 181) (see [84]). Again, this will not surprise anyone who walks through the winding streets of an Italian historic town center, like Via delle Volte in Ferrara (https://www.inferrara.it/it/p/80/arte-e-cultura/via-delle-volte, accessed on 12 March 2025). The term “enigma” is also mentioned repeatedly in De Chirico’s writings, which is not strange for the vision scientist. Michotte defined enigmas of perception as the presence of a visible although colorless percept (the *donné amodale*) [85] or perspective in outline pictures [86]. Finally, the assertion that visual reality is permeated with enigmas is what the Gestalt approach to perception has shown time and time again.

De Chirico’s paintings shape the following aspects that are important for Vision Science:

1. The what of the painter’s vision (qualitative perception in a subjective time–space order);
2. The global perception of ambiguity and apparent indecipherability of complex open sceneries, and their pervasive atmosphere [87] (like melancholy, sadness, fear, and estrangement);
3. Cultural influences that surface in every part of the percept: for example, the repeated presence of the Roman arch in many of his paintings, and its visible geometric characteristics of openness, repetitiveness, and line continuity.

With regard to the latter aspect, the line was a visual element that De Chirico considered fundamental (“the terrific power of the line and angles” [83] (p. 181)). The architectural composition of lines in a painting gave him the impression of structural traits of visual appearances (tectonics) that give visible shape to something (see [67,88]), that, when drawn, allowed his paintings to fly (see *L’énigme d’un après-midi d’automne* [The enigma of an autumn afternoon], 1910).

These aspects were enriched by several pictorial devices, such as impossibly long midday shadows or the artificial Figures that dominate many of his paintings with their combinations of Greek column pedestals, human torsos, and artificial heads (see *Le muse inquietanti* [The disquieting muses], late 1950s) (see Section 6). Parts, of what may once have had individual experiences (Greek columns, heads, etc.), are embedded in new wholes, destroying the Albertian idea of composition, and creating a disturbing tertiary quality. In fact, as Köhler observed, the way a certain part of the whole appears is influenced by the prevailing conditions in other parts of the whole [89].

How did De Chirico analytically proceed, and which intellectual and artificial tools did he use to give pictorial form to the awareness of the enigmatic subjective experience of visual configurations and the deep atmosphere of melancholy and estrangement they convey? How did he choose to render the what, the way, and the why things appeared to him, outside a lab?

It is worth noting that in his prolific writings as an art critic, journalist, novelist, and even autobiographer, we can find a detailed commentary on the subjective impulse that suddenly motivated his first metaphysical painting [83] (pp. 133–134), the need for recursive themes, the long periods required for each of his creations, and the meaning of his enigmatic painting. What matters, however, is what the viewer, even if unaware of these commentaries, immediately perceives when looking “into” his paintings [90].

## 5. The What: Visual Reality

De Chirico implemented a clear project of violating pictorial rules to enhance the subjective perception of reality. Compared with the experimental results obtained by Gestalt psychology, such as the demonstration of perceived phenomena like absence and nothingness, void and fullness (the fourth level of reality in Metzger [15]), his organization of the figure/ground went even further by visualizing the atmosphere (*Stimmung*) embedded within them in visual configurations. Atmosphere has been poorly analyzed by Gestalt psychology and experimental phenomenology (criticism in [91]), and, in addition to philosophy [92] and esthetics [93], this has also raised interest in psychopathology [94].

What De Chirico’s paintings show is not only that we do not have any direct access to physical reality, but that what we see and perceive are far beyond physics. “There is no matter in space,” as De Chirico wrote in a letter to Medardo Rosso. Perceived reality, what it *is* (τί ἦν εἶναι), from the point of view of physics, is extremely illusory, and the “invisibly present”, both at a perceptual and mental level [95], is a pervasive component of visual experience. However, there is much more than the phenomenology of appearances in De Chirico’s works. A visual experience of his paintings involves high-level mental processes triggered by literary sources, fragments of architectural shapes, symbology, and a knowledge of both art history and his personal memories (like the biscuits that are reminiscent of Jewish shops or the corporal rank insignia on the egg-shaped heads of a mannequin). These “signs”, as analyzed by Benussi [36], are responsible for high level mental processes that intervene as unreality realizers and reality multipliers.

So, how are the apparently inextricable primary and secondary dimensions of the psychic processes responsible for the subjective estrangement of the viewer visualized on the canvas, and what kind of vision information do they convey? An excellent analysis of how De Chirico was able to visualize the complexity of the visual perception of a natural scene, the rendering of newly acquired tertiary properties of appearances due to the configurational re-arrangements of the scene and moods, from melancholy to a thrilling sense of fear, can be found in Arnheim [66] (Ch. 5), who gives a detailed analysis of two of De Chirico’s most famous paintings from the same year (1914), *Stanchezza dell’infinito* [Tiredness of infinity] and *Mistero e malinconia di una strada* [Mystery and melancholy of a street].

A general characteristic of De Chirico’s paintings is that they give the viewer a first impression of a realistic scene, but, like *trompe-l’oeil*, one also feels immediately that something is not quite right. Notwithstanding the apparent sturdiness of the shapes, the global scene is not solid, and this gives the viewer a sense of being trapped in the geometric structure of a Necker cube or a Platonic solid.

Arnheim lists the series of contradictory pictorial devices the painter combined to trigger the perception of illusoriness in an apparently realistic scene. For example, in the painting *Stanchezza dell’infinito*, while the global composition is drawn with a central perspective, the statue (Ariadne) is placed on a cube following isometric rules, which gives the impression that it is projected rather than resting on the ground. The sides of the square also meet far above the railway and the tower on the horizon, so the viewer is caught between two possible visual sceneries:

1. The presence of an empty universe, which extends beyond time and space, and expands behind the railway and tower to the horizon with the square stretched on one side to create a void where no one could possibly be, and where the two lateral columns appear divided by a flat abyss.
2. At the same time, the viewer is offered a totally different scenario where the shape of the square is the reference scheme. Here, the colonnades that converge at the edge of the painting are impossibly shortened; so, if observed in isolation, they appear completely normal, except for the arch that is placed frontally on the far left.
3. Finally, the painting demonstrates the role of shadows, as the shadow of the colonnade on the right determines two vanishing points which are incompatible with the others.

So, what does the viewer really see (the what) when they look into the painting and how does it appear? In terms of awareness, the viewer’s viewpoint changes continuously, according to the visual instability and inconsistency of the geometric framework (similar to what happens with fourteenth century painters like Fra Angelico), and this apparent movement is embedded in the scene. So, the world we are seeing seems to be real, but it changes according to how we look “into” it and where we are led by the components that are balanced around a point somewhere outside the geometric center. We are, therefore, briefly confronted with an illusory, displaced universe of simultaneous contradictory appearances and meanings, which, for the vision scientist, is an issue of explanation.

A further aspect of De Chirico compositions that is worth noting, concerns the repetitive presence in his painting of certain items, like the square with a displaced statue in it, the railway, the tower, the clock, the glove, the ship’s bridge, the flags, the lighthouse (real nautical iconography) (see *Le muse inquietanti*, late 1950s, *La meditazione di Mercurio* [Mercury’s meditation], 1973), and his series of *Interiors* from the late 1960s. What matters here for Visual Science, as mentioned above, is how the repeated presence of a situational configuration of an object affects actual perception and determines the degree to which a visual object acquires new properties (see Section 1 above). For example, the lighthouse is so typical of marine settings and ship travel that it tends to reproduce this context and influence other perceived objects too. The simultaneous presence of a lighthouse in a very different and unexpected environment (a city center, for example) contributes to the illusoriness of the scene and the instability of its meaning. This point is interesting because, as Arnheim has observed [66], the situational configuration of an object also affects its way (*Weseneigenschaften*) of expressing character, ethos, habit, atmosphere (happy/sad, friendly/hostile, bold/fearful, passive/aggressive, and so on (see [15] (Ch. 2)). In the case of De Chirico’s painting, this is mainly exemplified in the polarity of sadness on the differential semantics scale [96]. We directly perceive tenacity, brittleness, grandeur, and many other attributes both in animate and inanimate things that, sometimes, we even lack linguistic descriptions for [97]. This is proof that the richness of vision is greater than any linguistic expression.

The second painting analyzed by Arnheim in detail is *Mistero e malinconia di una strada*, 1914, one of De Chirico’s most famous paintings, which he painted again and again in the 1970s with slight variations. As with the previous painting, the use of different spatial perspective schemes produces a conflicting view, because the apparent coherence of the scene is fragmented by a series of local contradictions. For example, if the viewer looks at the colonnade on the left, the colonnade on the right appears to literally sink into the ground. Vice versa, if we focus on the colonnade on the right, the horizon is hidden somewhere, below the painting itself. This produces an uncomfortable sensation, as the road that runs uphill by the side of a white colonnade seems to be leading a little girl who is heading up it to a potential leap into darkness. The mood triggered by the configuration is unsettling and close to fear.

So, what can we understand about the visual perception of scenes from De Chirico’s pictorial representation? We do not have direct access to distal stimuli, and our natural visual perception goes far beyond physics, as it is pervaded by so-called illusions and puzzles we have to solve, and framed by a series of objects held together by dimensions that underlie the organization of empirical factors in the field and enrich and modify what we are looking at. We have no power over these aspects, they simply pop up in the scene. The reality depicted by this metaphysical art is therefore a reality made up of appearances, co-determined by empirical and imaginative factors (deformations of the percept due to the situational focuses and mental frames of reference). In short, a reality that is very close to subjective experience. In De Chirico’s case, the structure of visual perception, much more than the mechanical logic of geometrical optics, follows what Kepes defined as the rule of “connectedness in meaning” [98], which corresponds to one of the principles of perceptual organization [99]. Moreover, a certain configuration, besides its visual appearance of color and shapes, possesses properties which cannot be retrieved by association or inferences from other sensory properties.

## 6. The How: Means to Illusoriness

Let us now summarize the factors responsible for the pervasive ambiguity of perception, the role they play in De Chirico’s metaphysical paintings, and the dimensions of visual awareness they display.

1. The presence of conflict between different perspectives in the same painting is related to the fragmentation of the center into several local ones. As Arnheim observed, “when centricity is overridden, its ineradicable perceptual presence overrides meaning… The psychological connotation of floating in the nowhere, in a space where no one place differs from the next, exerts its bliss or terror artistically when an anchor is explicitly represented as being denied” [70] (p. 107). The center, therefore, provides stability, which is exactly what is missing in De Chirico’s metaphysical painting.
2. The presence of objects that are out of context (like Magritte’s painting *La Bataille de l’Argonne*, 1959 or Dalì’s *Figueras*), such as statues with an open torso revealing an assembly of unexpected objects (see *Canzone meridionale* [Southern song], about 1930; *Gli archeologi* [Archeologists], 1940; *Oreste e Pilade* [Orestes and Pylades], 1960; *Oreste solitario* [Lonely Orestes], 1974) or De Chirico’s series of metaphysical *Interiors* (see *Interno metafisico con officina* [Metaphysical interior with workshop], 1969; *Interno metafisico con paesaggio romantico* [Metaphysical interior with romantic landscape], 1968; *Interno metafisico con testa di Esculapio* [Metaphysical interior with head of Aesculapius], 1969; *Interno metafisico con testa di Mercurio* [Metaphysical interior with head of Mercury], 1969; *Armonia della solitudine* [Harmony of solitude], 1976; *Visione metafisica di New York* [Metaphysical vision of New York], 1975; *Il grande trofeo misterioso* [The big mystery trophy], 1973; *Il sole sul cavalletto* [The sun on the easel], 1973); and the presentation of ancient architectural elements (Greek statues and columns, and medieval towers), alongside contemporary architectural elements (chimneys and railway stations). In fact, it is widely known that De Chirico had a significant influence on Magritte [100,101]. As already mentioned, the role of context and its violation is one of the main ways for an object to take on new appearances, which means acquiring new perceptual (tertiary) properties. For example, a statue in a Greek temple blends with nature and the beauty of the landscape. However, if it is detached from its context and trapped within the lines of walls, like the floor and ceiling of a museum or domestic room, it acquires new properties of estrangement and perceived solitude. De Chirico’s sensitivity in representing these states of affairs uncovers a further relevant aspect in visual perception. When the Venus de Milo or Hermes of Praxiteles statues are detached from their original location and placed in a museum, they are subjectively perceived as “dislocated”, which conveys, for a sensitive observer, and even more so for a cultured observer, a sense of estrangement. The “solitude” of a statue is not an anthropomorphic projection but a tertiary/expressive quality similar to the fact that a dark cloud is “sinister” in itself [14]. These expressive qualities relate to the positive/negative subjective impressions conveyed by the “stimuli,” and are nearly always present in perceptual phenomena in different sense modalities (including cross-modalities). These qualities have no physical basis, so it is impossible to measure them unless in terms of “more or less” along an adjectival continuum and using the Osgood semantic differential. Landscapes and open scenery as wholes also bear positive/negative qualities regarding the behavioral environment, and not exclusively the self, see [93]. In fact, the concept of *Stimmung* is closely tied to the expressive qualities of a whole given in a perceptual presentation. The concept of tertiary/expressive qualities also borders on the concept of affordance, which is often misunderstood in its original Gestalt meaning, being essentially related to motor action. Whereas Koffka’s conception of affordances (*Afforderungscharakter*) concerns how objects are perceived as having value and meaning as part of their physiognomic characteristics [14] (Ch. 8) [66]. Furthermore, perceptual aspects are acquired by objects when placed in an unnatural context (a statue sitting on a real armchair or leaning against a real window, or placed next to everyday objects like rulers, squares, biscuits, chairs, and so on [83] (p. 183ff) (and 383–386). These paintings are examples of the violation of the Requiredness principle analyzed by Köhler (like the fact that purple is necessarily a color between red and blue [50] (Ch. 3)). In each case, the presentation of objects that are out of context has an ambiguous effect.
3. The near omnipresence of inanimate things, like the ancestors of modern robots and avatars that, due to their over-large size and position in the center of the painting, behave as if they are in an imagined visual space ([56]. A lonely individual or, more often, a small, apparently lost human couple are sometimes placed in the squares (see *Le voyage émouvant* [The moving journey], 1913; *Piazza d’Italia con piedistallo vuoto* [Italian square with pedestal], 1955; *Piazza d’Italia con fontana* [Italian square with fountain], 1968; *Piazza d’Italia con statua di Cavour* [Italian square with statue of Cavour], 1974; *Piazza d’Italia* (*Souvenir d’Italie*), 1924–1925); *Il mattino delle muse* [The morning of the muses], 1972; *Le muse inquietanti* (late 1950s)) too, but, more often, humans are replaced by mannequins (artificial humanoids) and hybrids of statues and mannequins (see *Le duo* [The duo], 1914–1915; *Le vaticinateur* [The diviner], 1914–1915; *Orfeo trovadore stanco* [Orpheus, tired troubadour], 1970; *Il mattino delle muse*, 1972; *Le muse inquietanti*, late 1950s; *Ettore e Andromaca*, second half of the 1950s, and 1970; *Trovatore* [Troubadour], second half of the 1950s, and 1932/1972), triggering the emotional negative sense of an “uncanny valley” (see below Section 10).
4. Apparent movement, triggered by the presence of configurational elements (lighthouses, ship bridges, flags, etc.).
5. Long shadows in the full light of day.
6. The recursive presentation of the same objects with configurational and cultural values (statues, squares, ships, towers, arches, and fountains), and scientific measuring instruments (rulers, compasses, etc.) in different paintings. These elements appear in paintings of both interiors (room spaces) and exteriors (squares), as constantly returning presences of symbolic objects that continue parts of previously experienced configurations.
7. The reversal of interiors and exteriors that coexist in the spatial awareness of the viewer (see *Mobili nella valle* [Furniture in the valley], 1927–1930). This raises the issue of testing both the complexity of imaginary spaces with different complexities and different exterior/interior relations that are embedded in each other; and the role of the perceiver in these different spaces that are simultaneously in perception.
8. Expressive qualities endowed in non-inanimate things and events (see *Le destin du poète* [The destiny of the poet], 1914; *Composizione metafisica* [Metaphysical composition], 1950–1960; *Triangolo metafisico* [Metaphysical triangle], 1958; *Nature-morte. Torino printanière* [Still life. Spring Turin], 1914), which lie in the perceptual qualities of the pattern given by the coordinative field [102,103], and which are primarily responsible for the global atmosphere of the scene.
9. The complex geometric structure underlying these paintings as a source of their enigmatic atmosphere [104].
10. The “imaginary” nature of color appearances and the mastery of materials to produce them.

Regarding the latter, the numb appearances and shadows in De Chirico’s first metaphysical period are later totally supplanted by brilliant, saturated, vibrant colors that acquire a grounding role in vision (see *Interno Ferrarese* [Ferrara interior], 1916). As the years went by, in De Chirico’s painting, color prevailed over architectural form and became the “matter” of vision. Not surprisingly, De Chirico’s conception of the nature of color was influenced by the theories of Schopenhauer [105] and Goethe [106] (see [83] (pp. 391–403)).

De Chirico’s tireless research into how to render in painting the way things appear in subjective experience in imaginary spaces runs parallel to his masterful knowledge of physical and chemical artistic painting materials, especially his choice of colors. Highly informative research on the pigments used in De Chirico’s palette has recently been published [107] (see also [108]). This analysis has been made using X-Ray fluorescence (p-XRF) on 7 paintings at MoMA and 4 *veri-falsi* paintings from the Menil Collection (see results in Table 2, p. 6 of [107]. The outcome of this study has also been compared to De Chirico’s *Piccolo Trattato di Pittura* [Small treatise on painting] [109], his favorite color palette and his painting techniques (see Appendix A). Of note is the deliberate omission of vermilion in his metaphysical painting, as he considered it inadequate for melancholy contexts and is, therefore, only identifiable in a few paintings from his 1916–1920 period. Another point to be noted is his choice of different whites for painting different forms. For example, he uses a thick, opaque lead white for architectural forms and sculptures, and a clearer, pale zinc white for non-architectural forms, such as clouds and mannequin heads. Interestingly, in his paintings from the 1920s, light emerges only in patches around the legs and arms of the figures (see *Nus antiques* or *Donne romane* [Roman women), 1927; *Archeologi* (Archeologists], about 1927; *Gladiatori* [Gladiators], 1928). His veiling technique is curious too, as it makes shades brighter and more transparent, and allows pure white (like the appearance of white light in Raphael’s *Transfiguration* or Correggio’s *The Night*) to be obtained using a base of zinc white with tiny layers of pastel colors, from which the white emerges. The last part of his *Piccolo Trattato* focuses on absolute white (the what) and veiling (its way of appearing). In this way, the geometric layout of his first metaphysical period undergoes a transformation towards a deeper understanding of the nature of visual appearances that consists of a unity of color and light qualities [110].

## 7. Empirical Analysis: La Matinée Angoissante

An interesting aspect related to the visual perception of appearances is De Chirico’s use of color for rendering three-dimensionality, relief, and distant light contrasts along the horizon that are perceived as separating the ground from the sky. To visualize the latter, De Chirico uses yellows and blue greens to render the experience of the color bands on the horizon and their sense of space and order of depth (see *La matinée angoissante* [The anguished morning], 1912; *Piazza d’Italia con Arianna* [Italian square with Ariadne], about 1935/40; *Malinconia di Arianna* [Ariadne’s melancholy], 1912). The use of these colors appears to be connected to the perception of open sceneries. Several studies on the artworks of individual painters [111], and more recently in the field of automatic image interpretation [112], which involves extracting bands of color [113], have revealed the presence in perception of generic templates for landscapes. For example, in the dataset of retrievable images, yellow-blue and black-white gradients correspond to the low-high position. In other words, a color scheme seems to exist, ranging from yellow-brown through green-yellow, to whitish blue and deep blue, which governs the visual experience from bottom to top in the experienced visual field. A recent experimental study on this topic [114] has confirmed that viewers rely on these generic landscape templates. The textured images used for the test stimuli (made of convex polygons randomly filled with paired colors that gradually change to form a fuzzy ragged edge) look like an abstract painting. A total of 264 stimulus pairs, including yellow, yellow-green, green, cyan, cyan blue, blue, blue-magenta, magenta-red, red, and red-yellow (orange) were selected from the set and randomly presented in equal numbers. The task given to the participants was to evaluate which side of a square was closest using a series of corresponding left, right, up and down arrow keys. The results show that differences in tone evoke different perceptions of depth order in most observers: the lower side of an image is frequently seen as being closer than the upper side, and warm colors move forward while cool colors move back (see [115,116,117]).

De Chirico’s use of yellows and blue-greens to render the experience of color bands on the horizon in his landscapes, as well as their sense of space and order of depth are therefore very interesting, and in line with the findings of visual color schemes, especially because of the unusual dark surrounding areas in his metaphysical paintings. Hence, the decision to analyze one of the most famous paintings in De Chirico’s metaphysical period. I hypothesized that De Chirico’s painting could provide further understanding of the structure of natural visual templates in open scenery, hue contrasts in perceived spaces, cast and own shadows, perceived three-dimensionality, the interaction between hue and lightness, and the global expressive atmosphere of the scenery.

The phenomenological analysis of De Chirico’s painting, *La matinée angoissante* [The anguished morning] (1912), on display at the Museum of Modern Art in Rovereto, Italy (MART, https://www.mart.tn.it/opere/la-matinee-angoissante-87282, accessed on 12 March 2025); a high resolution image is visible in the Supplementary Material) allowed me to focus on the area of the horizon bands in the canvas and analyze the contrast between the yellow–green–blue and brown–purple colors chosen by the painter using NCS (Natural Color System) notation cards. I chose the NCS color system because it allows the similarities and differences between colors to be assessed according to their visual appearance only. Conceived and built on the Hering theory, the NCS methodologically requires colors to be assessed subjectively; it is, therefore, the method best suited to a phenomenological approach to studying color. As a reference system, Refs. [118,119] NCS indicates the inter-relationships between colors in a spatial form, as they are qualitatively perceived by humans.

To conduct the analysis, the painting was moved specially to a separate environment outside the Museum and with no visitors present. The perceptual measurements of the painting were made by designate observers.

**Figure 1:**
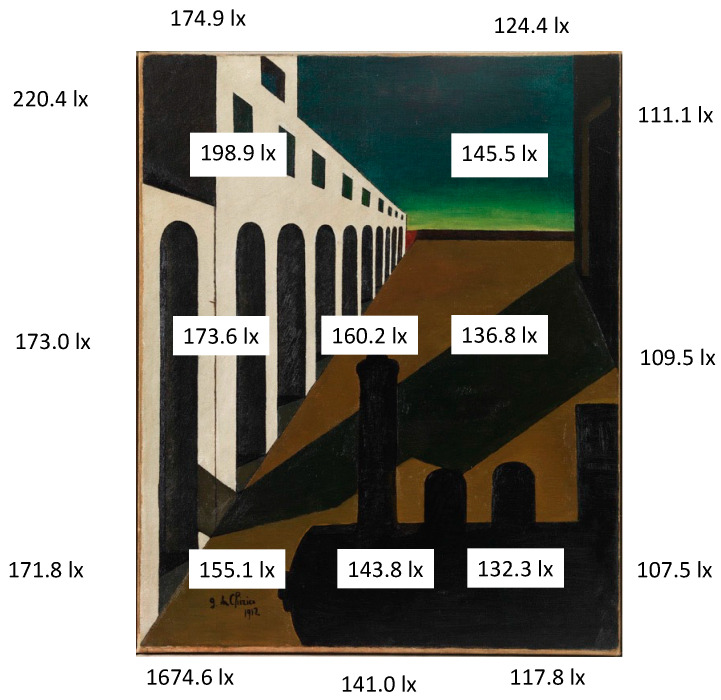
The lighting measurements taken of the painting.

**Figure 2:**
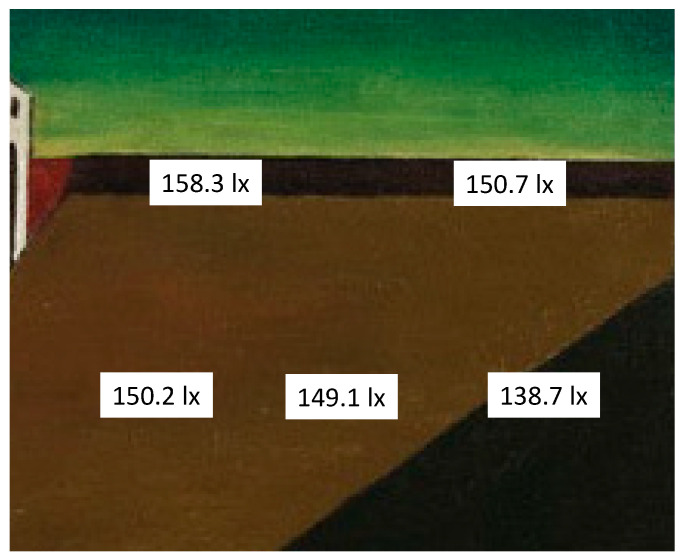
The lighting measurements taken of the bands on the horizon.

The ambient lighting, the light falling directly on the painting and its background were measured with a luxmeter (Illuminance Meter T-10, Konica Minolta) (see Figure 1 and Figure 2). Lighting measurements were also made all the way around the painting (right, left, below, above and with the instrument held parallel to it) and on it too (with the instrument held parallel to it in the closest, safe position: a distance of 7–8 cm). The white cap on the meter head collects light from all directions and directs it to the sensor. The painting was not uniformly illuminated as the light source was on the ceiling to the right of the painting (on the left of the person observing it).

As shown by the illuminance measurements carried out with a luxmeter, the painting was placed in a spatial position illuminated by a D65 source located above and to the left, and therefore the external lighting of the painting and the virtual internal one corresponded. The result is that the painting is not uniformly illuminated, but the light is distributed on its surface with a continuous gradient from the highest level in the top left corner to the lowest in the bottom right corner. As the physical variation in lighting on the different areas of the painting is gradual, without any “jump” in brightness being seen between two nearby points, the colors are not altered by this non-uniform lighting. In fact, not only are no differences in illumination noticeable, but also no differences in clarity of the illuminated color are detected between any two nearby points, because they are “below threshold” (see Retinex Theory).

**Figure 3:**
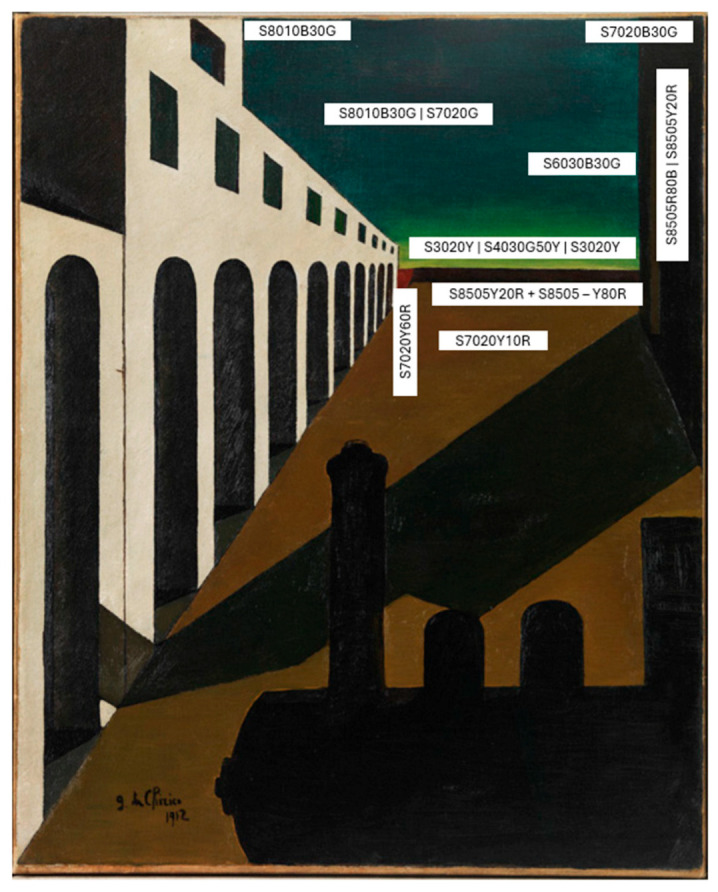
The color notation analysis on the horizon bands.

For the measurements of the color bands on the horizon, I relied on the two NCS Atlases. They were placed on a trolley in front of the painting, which made the comparison operation easy. The paper cards, potentially resembling the color bands on the painting, were taken from different sheets in the two Atlases and compared with them one by one. Care was taken to keep the cards in an accurate vertical position in relation to the canvas when making the comparison. The following results were obtained (see Figure 3).

**Figure 4:**
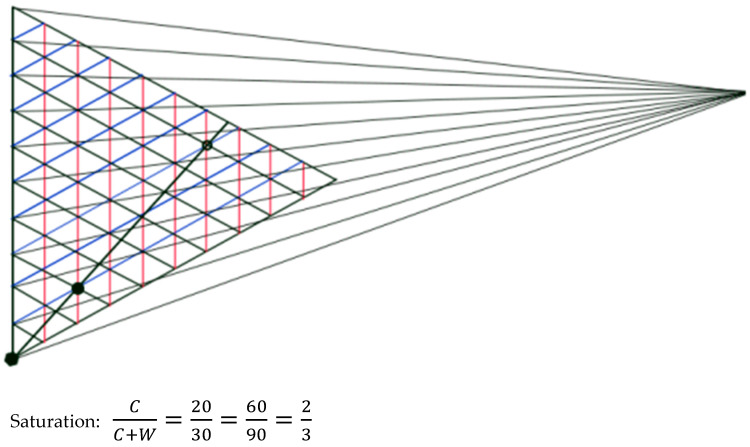
The two small black shapes (the hexagon and circle), which show the position of the open air and shadowed colors in the NCS triangle, determine the saturation degree of the two colors. The light circle is shown for explanatory purposes. It is on the equal saturation line determined by the previously mentioned colors, and, for this reason, has the same saturation, which can be calculated according to the NCS formula S = C/(C + W). Colors with the same saturation give the impression of having surfaces of identical color but are in different planes and therefore under different illumination. Equal Blackness lines are thin and in black; equal *Whiteness* lines are in blue; equal Chromaticness lines are in red (vertical); and equal Lightness lines are in magenta. Constant lightness lines converge outside the triangle at a point typical of the hue.

The colors in this painting are generally not very chromatic (see the NCS notation in Figure 4), as their chromaticness is, on average, just 10% of the total color attributes.

However, due to some of the interesting characteristics (graduated shading and shadows), indicated below, the painting appears dark but very chromatic.

## 8. Discussion: Relevant Outcomes

Thanks to De Chirico’s masterly use of color, lighting, and shadows, this empirical analysis shows how relevant his art is for various aspects of Vision Science; more specifically as follows.

(i) Cast and own shadows

In this picture, there are mainly only cast shadows, except for the dark inner vertical side of the arches which transmits a powerful impression of intense illumination outside. At the same time, the 3D appearances of the arches appear accentuated by the significant contrast between the lit and shadowed sides of the arches.

In the lower left part of the picture, the external surfaces of the arches, which are well lit elsewhere, are covered by a cast shadow whose reduction color (a color which is seen in isolation) is very similar to the inner dark floor of the arches. Nevertheless, the colors of the two areas are differentiable because under the arches, the color appears opaque, while outside the arches, it has a double appearance, that of the underlying wall and that of the superimposed shadow.

It has been shown [120] that the color of a surface in the open air and that of the reduction color of a shadowed area of the same surface have the same NCS saturation. If we consider the color of the main floor and the reduction color (a color seen through a pierced surface) of the shadowed floor, they can be considered as belonging to an equal-saturation line converging on the black point of a NCS triangle (see Figure 4). The calculated saturation is quite high, two thirds, and gives the impression that the floor color is quite chromatic, more than when it is observed without the shadow. Probably, color contrasts play a role here too.

(ii) Graduated shading and the interaction of hue and lightness

In the upper part of the picture, a downward gradient from dark to a light greenish color creates two double visual effects: a strong hue and lightness contrast with the dark reddish color depicting the floor, and an impression of brightness in the adjacent area (see [121]).

The color context here is a combined effect of hue and lightness, due to the opponency of green and red, while the lightness contrast is created by both the achromatic (luminance) contrast between the two areas and the warm and cold appearance of the two color groups that have very similar NCS nuances.

What remains difficult to decipher is the black stripe dividing the upper from the lower part of the picture, and even less understandable is the red area at the far end of the arches. Probably, these are just pictorial devices to enrich the color composition.

(iii) Verticality, horizontality, and color surfaces appearances

The black locomotive in the lower right part of the picture seems to be vertical unlike the shadow on the ground, and even if the black color used in the two areas is quite similar, they appear very different in terms of color and spatial orientation.

(iv) Lighting and atmosphere in landscape templates

The color of the bands highlights the ecological validity of generic landscape templates in much darker scenery too, as the contrast between light and darkness increases the visual effect of brightness in the upper horizon band (which is a sort of Baroque luminous “line”).

Unlike the above-mentioned study [114], the “stimulus” here is a real painting (as opposed to dataset or abstract images). This is more like the observation of a real scene, and it is therefore more phenomenally perceptual. Furthermore, the color analysis method used is different and closer to the subjective perception of color. These choices were neither casual nor arbitrary, but simply better suited to the empirical methodology of experimental phenomenology applied here. Finally, the given of a very dark environmental landscape in the painting, which increases the perceptual brightness of the upper horizon bands added further dramatic information regarding the perceived atmosphere of the whole landscape as “gloomy” (for the expressive quality of moods, like gloom or sadness in lighting, see [122,123]). This was De Chirico’s exact intention (see the same issue in an even more multi-stable perceptual framework, *Le voyage émouvant* [The moving journey], 1913).

(v) Connotative qualities of colors

De Chirico’s study of visual appearances shows the choice of certain colors for their expressivity, like dark colored areas to express melancholy, sadness, and fear. The same effect is produced by the deep dark green used to depict the water in the ground-based (coffin-like) trapezoidal fountain in *Les plaisirs du poéte* [The poet’s pleasures], 1913, and the shadow on the right, that invades the brightly lit square. Furthermore, in the painting De Chirico uses the warm and cold appearance of the two color groups, which have very similar NCS nuances. The yellowish colors (in the upper part of the picture) are lighter the while reddish colors (in the lower part of the picture) are darker, see [115]. This difference in lightness which corresponds to the natural lightness of the hues seems to be why the relative surfaces appear on the same plane, see [116]. On connotative warm–cool gradients of colors see [124].

The empirical analysis performed on De Chirico’s painting should be further subjected to experimental verification following the methods of experimental phenomenology (for the methods see [125,126]).

(vi) General validity of results for other art works

Besides the information provided on visual phenomena, the results obtained here can be used to analyze the artworks of other painters too. In fact, a similarly disturbing atmosphere of loneliness, estrangement, and unease is masterly conveyed by Hopper’s paintings (https://www.wikiart.org/en/edward-hopper, accessed on 12 March 2025). This, too, is consciously created through a careful distribution of light and shadow in the faintly surrealistic interior and exterior spaces depicted. The atmosphere of *Rooms by the sea* (*Alias the jumping off place*) (1951) is rendered positively frightening by the looming presence of the sea mass against the interior floor of a quiet luminous room. Another example is the reversal of interiors and exteriors that, like in De Chirico (and Magritte as well), coexist in the viewer’s polyphonic spatial awareness (see the whole series of rooms by the sea and other paintings).

Further instances of the moods that De Chirico literally “painted” can be found in the surreal, negative, emotional liminal spaces of empty, digital esthetic interiors (https://en.wikipedia.org/wiki/Liminal_space_(aesthetic) (accessed on 12 March 2025) [127]. Most importantly, the disorienting atmosphere of De Chirico’s painting permeates the research conducted into artificial humanoids by enhancing the opposing feelings triggered by the increasing resemblance of artificial agents to humans (the “uncanny valley” phenomenon in robotics (see https://spectrum.ieee.org/the-uncanny-valley, accessed on 12 March 2025), and [128]. This constitutes a real psychological jump into the contrasting dimensions deep inside the imagination that floods our natural perception. Once more, the psychological literature (see for example [129]) and artworks by the artistic avantgardes of the twentieth century are important sources of inspiration and enlightenment for contemporary issues in vision research, computer science, and neurophysiological correlates (see for example [130]). De Chirico’s art consciously prefigured this situation by incorporating mannequins and other out-of-place objects to deliberately create emotional disorientation and uncertainty.

## 9. Conclusions: The Role of Imagination in a Visual World of Appearances

In this study, I have discussed a series of issues still that have not been properly addressed (like the pervasive role of imagination in perception) or clarified (like the relationship between primary and secondary processes) in Vision Science. This paper presents an experimental phenomenological approach and framework and includes descriptive and empirical sections. As already mentioned, adopting the legacy of Gestalt psychology and experimental phenomenology, this paper considers works of art as a bench test for the study of perception. In this regard, De Chirico’s metaphysical painting has been chosen because it crosses several levels of psychic processes, the perceptual awareness of ambiguity and atmosphere in the global visual scene, and the grounding and pervasive role of imagination. In short, metaphysical painting provides a deep understanding of our subjective perception of the environment we see and its layered structure.

More specifically, with regard to the relevance of the results obtained for extant scholarship, the analysis of a number of De Chirico’s paintings (i) shows the co-existence of primary and secondary processes in seeing phenomena; (ii) highlights the behavior of past experience as a product of field organization; and (iii) underlies the role of imagination in making appearances visible (at different levels of reality), including our perception of emptiness and moods. All these aspects are embedded in these paintings, just as they occur in natural perception. Furthermore, De Chirico’s painting provides information about the research questions raised in the introduction, i.e., what we see, how we see, and why we see what we do in seeing.

As for “the what”, De Chirico’s painting shows that we perceive visual appearances as “real” in different degrees, located in subjective space, time, and movement and endowed with contrasting emotional features. Appearances can change according to internal and field relational conditions. Tertiary qualities are also perceived as embedded in appearances [3,14], as are values which are objectively and necessarily included in the context [50]. However, the real relevance of De Chirico’s art to Vision Science is his ability to assemble and visualize different layers of visual phenomenal presence in the same painting, so they are perceived simultaneously by the observer. De Chirico, therefore, succeeds in shaping what can be defined as the “logic of imagination” that operates in the perceptual experience of a visual, natural or pictorial configuration.

As for “the how”, De Chirico offers several visual examples of the intertwined presence of primary and secondary processes in the experience of a scene, and to achieve this in his paintings, he uses a series of enigmatic and often disturbing configurations.

Moreover, his scholarly mastery of colors, shown by the empirical test conducted on *La matinée angoissante*, confirms the presence of templates in vision, provides information about the contrast between hues and subjective perceived illumination in a scene, and the resulting atmosphere. The colors and dark atmosphere of the scenery depicted, in fact, combine to enhance the tertiary qualities that characterize the perceived ambient illumination [123].

As for “the why”, De Chirico’s painting raises what I define as an unavoidable mind-set reversal in Vision Science, i.e., the need to start considering subjective vision beyond the never-questioned grounding of physics [7]. This reversal implies considering new dimensions of seeing, which are often neglected or limited to the field of esthetics (conceived as a theory of pleasantness or of beauty), new methodologies, new explanations of visual perception, and new models for representing this complexity.

The settings of De Chirico’s metaphysical paintings and the relevant appearances he repeatedly shapes (the lighthouse or square, for example, which have both a behavioral and an expressive character) show how imagination deeply permeates our encountered visual reality in a way that is as admirable as it may be shocking. In line with the “imagination thesis” [131], which indicates the inner relationship between imagined pictorial visual space and subjective visual perception, what the viewer achieves here even more radically when looking at a picture is the awareness of being immersed in the pervasive field of imagination.

The results produced by this study have led to a consideration of what may be the experimental tools needed to verify the above-mentioned aspects, both in terms of the methodology to adopt and the type of laboratory to conceive and construct. In fact, the question of the kind of laboratory required to perform experiments is a crucial issue in verifying what happens in the subjective vision of open scenery.

## 10. Future Directions: Laboratories for Visual Imagination

Imagination in science has been studied principally from a psychophysical [132] and neurophysiological viewpoint [133,134,135,136] (for a review of the state of the art, see [33,137]; or, from a top down account, ref. [32]).

What I have analyzed in this paper, on the other hand, is the root process of imagination in generating appearances in awareness, and its central place in subjective visual perception. To be blunt, I would borrow Brown’s words that “most if not all perceptual experiences are infused with imagination”; and I agree with his observation that we currently lack a framework for debating this hypothesis. It is, therefore, in the interest of all vision scientists to help construct this framework, in which works of art can be an incredible source of inspiration. Besides the availability of well-known equipped laboratories, now we have newer technologies at our disposal for analyzing visual perception both in natural and pictorial space. The subjective awareness of immersion in seeing, for example, is enriched and augmented to offer significantly more information than is normally available [138]. Augmented Reality (AR) and Virtual Reality (VR) are two important research fields for testing the realm of appearances and the stratified levels of awareness (“presence”) that Metzger describes, and which De Chirico offers multiple examples of in his metaphysical painting. AR and VR can also overcome the limits of questionnaires and interviews that are often used to accompany and support neurophysiological research in vision as descriptive tools for subjective experiences [139,140].

However, these new tools that allow perception in a computer-generated 3D reality need to overcome a series of current technical limits. With VR, for example, these include obtaining a good sense of haptics and replacing the clutter of a head-mounted display and projection-based LED screen with good 3D sound and good interaction (like movement tracking, including finger/gesture tracking or even facial expression recognition), and granularity of visual representation. Many other issues arise from the use of different VR technologies, and their interaction with other research fields, such as computer graphics and human machine interaction. The thorniest problems encountered by VR, however, are how to render the subjective dimensions of experience, such as space, time, texture, color and light appearances. This implies an unavoidable step into experimental phenomenology research. In fact, once the issue of comparing the physical environment to digital images is solved, VR must also address the comparison between physical reality and subjectively perceived reality. For example, adopting a correct methodology for implementing the subjective experience of color requires considering the phenomena of color and light appearances, such as color contrast, brightness, natural lightness [141], and their connotative properties [115,123], its relationship with the background [142] and depth [9,143], and, more generally, the vast realm of color “illusions” [144].

An excellent analysis of the multiple issues to be faced and solved in this field is Stahre [145]. A similar complexity arises in modeling 3D perception and depth, when, in addition to metrical distance, the subjective dimension of the experience of remoteness also needs to be considered [146]. In short, one of the current challenges of VR is how to meet the requirements of physical realism which, up until now, has meant basing computations on physical parameters alone. In fact, people working in Virtual Reality still speak in terms of “illusions” for percepts which do not conform to physical stimuli. These include the “place illusion” and the “plausibility illusion”, i.e., illusions which give the impression that virtual situations and events are really happening [147]. In any case, these new tools could be used to devise or improve experiments for analyzing the subjective dimensions of perception. In this field, VR has already gone a long way to verifying phenomena, like the Kappa effect, and confirming it to be a mode of phenomenal awareness and a good laboratory for experimenting [148] and implementing it in AI. VR could also help us to better understand the experience of several levels of “illusoriness” of the reality we perceive. A few examples of these implementations, related to artwork, already exist, even if they have different purposes. For example, Sotheby’s has collaborated with the FGreat studio (https://fgreat.studio/, accessed on 12 March 2025) to create a 360° immersive experience of Surrealist paintings. There is also Damjan Jovanovic’s work on the complex geometries of Duchamp’s *The Third Glass* as a virtual reality-experience. And a virtual experience of Dali’s *Dreams* can be seen using an Oculus Rift headset. Similar creations exist for De Chirico’s paintings, like *Malinconia di una strada* [Melancholy of a street] and other related Dali’s paintings like *The persistence of memory*, and *Reminiscenze archeologiche dell’Angelus di Millet* [Archeological reminiscences of Millet’s Angelus] (1933–1935). These are works that are in some way related to Böcklin’s *Insel of the Dead*, which De Chirico admired both for its poetical and fantastical aspects and the quality of its painting. Other interesting tools can be found in the ongoing development of new forms of 3D geometry in the field of computer graphics. Fovotec (www.fovotec.com, accessed on 12 March 2025), for example, aims to measure and capture the subjective experience of seeing in the natural environment through a new form of nonlinear “natural” perspective.

These creations may well be effective tools for analyzing, describing, and explaining the role of imagination, odd perceptions (for example, after the experiment on the Kappa effect, a complementary experiment should be performed on the Tau effect [149]), and the varying degrees of ambiguity in the visual perception of open scenery. AR and VR could also help conduct a “real psychic analysis” [36], by combining certain functions with an analysis of the various states of absence, negation, assertion, firmness, melancholy, sadness, disorientation, and estrangement perceived in a configuration. In other words, these new tools can conduct serious analyses of the nature and comprehensive role of imagination in perceptual visual presentations. Finally, it would be interesting to design experiments for analyzing the kind of ambiguous space in which De Chirico’s sceneries are played, be that a Necker cube or one or more Platonic solids.

As mentioned, complementary analyses in neuroscience have recently identified the neural correlates of both imagery (based on past experience, from [133] onwards) and imagination (psychic processes that emerge inwardly without being related to something already experienced [150]). More recent studies have focused on the relationship between seen and imagined objects decoded by a pattern of activity in the ventral processing stream [134]. They have also reproposed the concept of mental imagery as a simulation of perception [151] or of the absence of bottom-up processes during imagery [137] and the vividness of imagery ranging from aphantasia to hyperphantasia [33]. On the other hand, Brown’s [152] analysis suggests that, from a neurological perspective, imagination seems to emerge in a neurobiological context related to self-awareness [152]. This would suggest that neural circuits can combine concepts and images with a direct perceptual origin in original ways to produce new images that enrich and modify the experience of a certain phenomenon. Most interestingly, the processes underlying imagination directly refer to phenomena which are stimulus independent.

Notwithstanding their differences, the results obtained so far in different research fields suggest there is a continuity between the different dimensions of awareness. From this viewpoint, while neurophysiological studies focus on the perception of spatially distributed entities like complex open scenery, and several cognitive functions have been attributed to the network of a scene (like scene recognition, spatial perception, spatial navigation, and so on) [153], it would be interesting to find the neural mechanisms underlying the different levels implied in the subjective perception of the enigmatic open sceneries depicted in De Chirico’s paintings.

However, despite all this, most neurophysiological studies concern imagery in its currently established meaning. So, for the time being, there is no definitive neurophysiological explanation of the role of imagination as discussed in this paper, i.e., as a process that roots and saturates perception. Consequently, it would be premature to discuss the correlation between neural activity and subjective perceptual visual experience. The results we have obtained so far allow us to formulate a hypothesis that favors an experimental phenomenological approach to vision as the first step in an investigation of neurophysiological correlations.

## Data Availability

Data is contained within the article or Supplemental Material. The original contributions presented in this study are included in the paper/Supplementary Materials. Further inquiries can be directed to the corresponding author.

## Supplementary Materials

The following supporting information can be downloaded at: https://www.mdpi.com/article/10.3390/vision9020025/s1, Figure S1: Credits: Mart—Photographic archive and Media library.

## Appendix A

### De Chirico, an Idiosyncratic Avantgarde Painter

De Chirico (Volo, 1888—Rome, 1978) was an idiosyncratic painter with a surprising balance of classicism and avantgarde. An active participant, exponent, influencer and then critic of the 20th century *avant-garde*, Giorgio De Chirico created eccentric kaleidoscopic sceneries where fragments of Cubism, Futurism (see Marinetti’s 1910 Paris *Manifesto*), Surrealism (Magritte), Dadaism (see Tzara’s 1918 *Dada Manifesto*), and Neue Sachlichkeit (Dix, Grosz) combine to produce structures that are not always symmetrical. With controversial opinions on most avant-garde movements, he was often arrogantly critical of what he considered their limitations (essentially their rush to abandon the expertise of classical drawing and a knowledge of painting and pictorial tools). The only close artistic relationship of his metaphysical painting that he recognized was with his contemporary Carrà [154] (see [83] (pp. 257–260)) and its affinity with the works of other Italian colleagues, such as Soffici, Papini, De Pisis, and Morandi. Vice versa, he never accepted the rejection of the tradition that had fueled the Cubist and Futurist rebellion or Impressionism, which, in his mind, consisted in the loss of pictorial sense [83] (pp. 183–184). A recent edition of his writings (1910–1978) (2814 pages!) is a detailed and excellent source of information on his life and work [83].

It is worth recalling the opinions of some of his contemporaries on his artistic production, to underline why his creations are of such interest to Vision Science. Apollinaire, in 1912, immediately pointed out «The strangeness of the plastic enigmas offered by Mr. De Chirico». Breton, in 1920, defined his paintings as «A true modern mythology, whose mind lends itself perfectly to reviewing the sensitive data of time and space.» Longhi, the harshest critic of De Chirico’s “Metaphysical art” and avantgarde exclaimed, “Too many mannequins!”, which earned De Chirico the nickname of *Homo orthopedicus*, and “Too much literature!”, insinuating that his paintings looked like literary documents. His use of «out of date» artificial instruments was also criticized, and most despised of all were his depictions of enigmatic configurations and the aura of mystery his paintings trigger (something that De Chirico shared with Böcklin, whose influence can be found in his paintings [83] (pp. 237–242)) (see *L’énigme de l’oracle*, 1910). These judgements can be seen as just the typical excessive attacks of an art critic, if it were not for the fact that they capture, albeit negatively, the essence of De Chirico’s painting.

Amongst his other abilities, and in accordance with his artistic conceptions, De Chirico was an expert in the chemistry of materials, and worked strenuously to restore both a knowledge and the techniques of classical painters [83] (pp. 208–212) (see the *Piccolo Trattato di Tecnica Pittorica* (1928) in [83] (pp. 391–438)). In the 1940s, he developed a painting technique (emulsion) that gives the pictorial surface a brilliant sheen by applying liquid pastes that create rich veil effects and dark shadows (see [83] (pp. 208–212)). This technique marks a change with the previous techniques he adopted that focused on geometry and perspectives. He later produced emplastic oil, a more elaborate technique than emulsion, which he used, for example, in his *Autoritratto nudo* [Nude self-portrait], 1943. De Chirico’s favorite metaphysical palette consisted of lead white, zinc white, bone black, vine black, burnt Sienna, burnt umber, van Dyck brown, Morellone red, red earth, vermillion, carmine lake, yellow ochre, Naples yellow, chrome yellow (lemon and orange), “brilliant” yellow, emerald green, Veronese green, green earth, “mineral” blue, Prussian blue, cerulean blue, and cobalt violet [107] (pp. 2–3).

Remarkably cultured, as shown by his writings [83], De Chirico is often accused of crowding too many literary references into his paintings. Here, I will focus on just two main theoretical sources related to his metaphysical production. On one hand, Schopenhauer [155], whose idea of foreboding influenced the permeating illusory landscape and atmosphere of his paintings, and his theory of color; and on the other, Nietzsche [156], see [157], who, as a result of the cultural environment of the 20th century, played a specific role in the thematic conception of his paintings (see *La meditation autumnale* [An autumnal meditation], 1912), and their almost infinite repetitions over the years. An author who is less quoted, but has analyzed the impression of disorientation and fear that pervades his metaphysical painting, is Weininger (see his “metaphysical geometry”, 1904/2001; see [83] (pp. 180–182)). In any case, it would be out of place here to analytically mention all the many influences that permeate his paintings [158] and catch the eye of the cultured observer (see *Trofeo con la testa di Giove* [Trophy with the head of Jupiter], 1929–1930).

De Chirico produced a higher-level of abstract art than cubism and impressionism, which is somehow close to symbolism (like the works of Moreau, Redon, Carriere, Böcklin, von Stuck and Gauguin (see Jean Moréas’ 1886 Manifesto published in *Le Figaro*)), with which he shared several thematic aspects. These include the idea that visual things can represent other things, and that, rather than representing physical reality, art shapes concrete and abstract concepts and emotions. He also shared with symbolism the means for conveying an entire concept, such as his references to Greek mythology, the presence of monstrous, hieratic or impossible, artificial Figures (mannequins), and his unsettling moods. Regarding the latter, De Chirico is very close to Gauguin’s idea that explanatory attributes—known symbols—congeal the canvas into a melancholy reality (see his *Where Do We Come From? What Are We? Where Are We Going?* (1897–1898). As to the role of shadows, doubles, and self-portraits in his painting, the influence he had on Warhol’s serialization of his *Shadows* (1978–1979) is widely recognized (https://www.guggenheim-bilbao.eus/en/learn/schools/teachers-guides/shadows, accessed on 12 March 2025). In this context, the transparency of the shadows should also be considered.

What De Chirico essentially does, through pictorial awareness, is trigger estrangement and melancholy by connecting them to both natural and cultural codes. This should not come as a surprise, as artistic production in many other Western and Oriental traditions often mixes natural and cultural codes. Think, for example, of Japanese gardens, where the way rocks are arranged can symbolize written Chinese letters, turtles, cranes, boats, gourds, mountains, or islands that call to mind legends or moral tales [159].

Shortly before 1919, de Chirico revealed his intention to produce a deeper, more complex, more complete art, essentially “a new metaphysics, and a new melancholy”, traits he saw as being closely connected [83] (pp. 158–161). After a long, self-coercive interval of elaborating classical drawings and paintings to refine his own expertise in drawing and color, and a period of neo-baroque painting, he expanded and repeated the grounding elements that recur in his paintings until the end of the 1970s, a time which he defined as “More metaphysical art”. For the purposes of this paper, we have mainly considered his first period of metaphysical art (1910–1912).
