# Supplementary material for: μετὰ τὰ ϕυσικά: Vision Far Beyond Physics
Source: Vision (Basel). 2025 Mar 26;9(2):25. doi: 10.3390/vision9020025 (PMC12015877; doi:10.3390/vision9020025)
Supplement: Supplementary file 1 [file vision-09-00025-s001.zip › vision-3483522-supplementary.pdf]

Figure S1. Credits: Mart - Photographic archive and Media library.

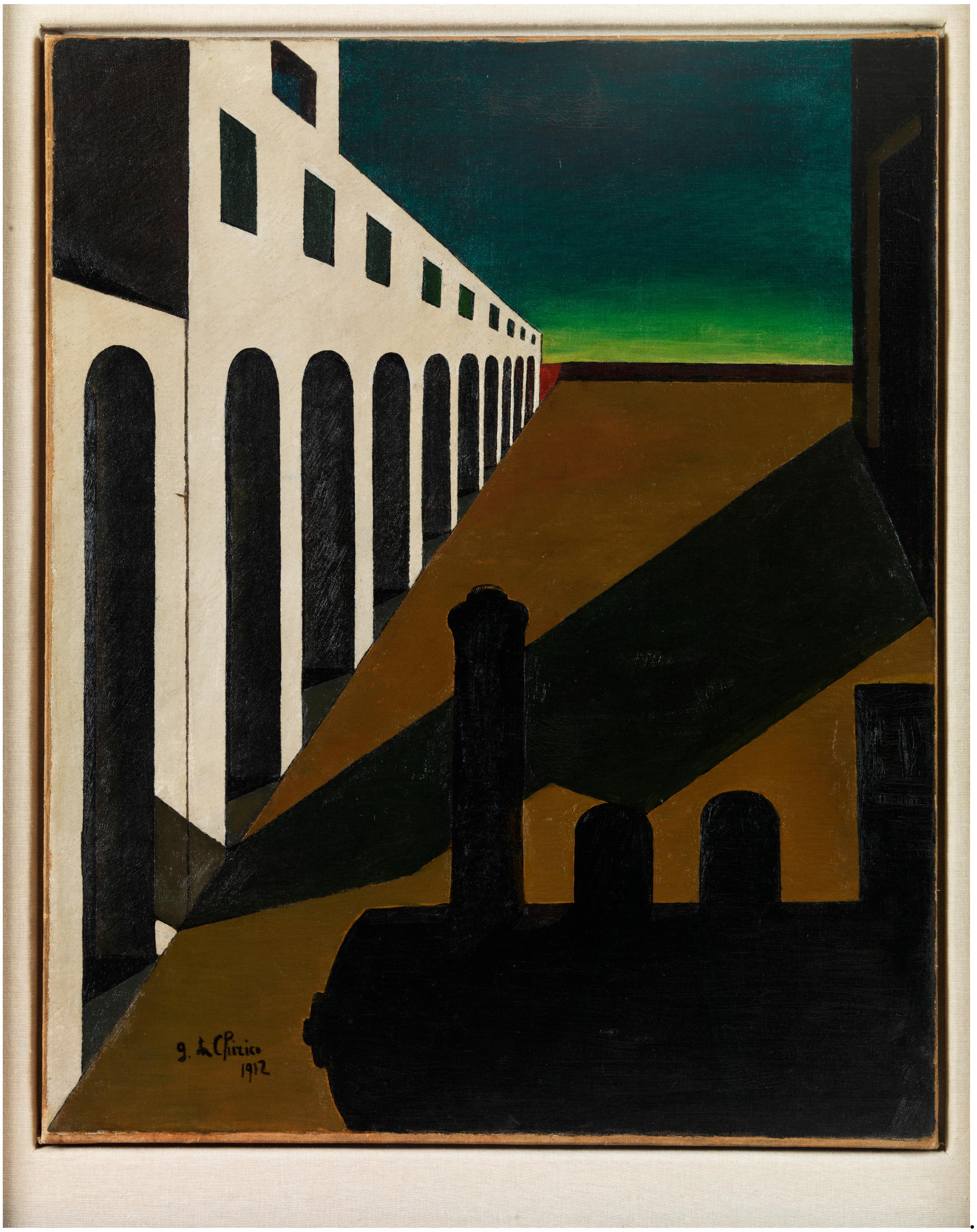

Giorgio de Chirico (Volo, 1888 - Roma, 1978)

La matinée angoissante, 1912

olio su tela, 81 x 65 cm;

Mart, Museo di arte moderna e contemporanea di Trento e Rovereto Collezione VAF-Stiftung
